# Evidence for dystonia reduction in cerebral palsy remains limited

**DOI:** 10.1111/dmcn.15923

**Published:** 2024-04-19

**Authors:** Daniel E. Lumsden

**Affiliations:** ^1^ Complex Motor Disorder Service, Children's Neurosciences Evelina London Children's Hospital London UK

## Abstract

This commentary is on the clinical practice guideline by Fehlings et al. on pages 1133–1147 of this issue.

It is now widely acknowledged that dystonia is a common finding in individuals with cerebral palsy (CP), both within and beyond dyskinetic CP. Involuntary movements and posture resulting from dystonia can cause pain and interfere with function and the delivery of care, necessitating in many cases management with pharmacological or other interventions. In 2018, Fehlings et al. reported on the systematic review of available data which had informed the development of the American Academy of Cerebral Palsy and Developmental Medicine (AACPDM) Dystonia in CP Care Pathway.^1^ Arguably the most significant finding of this review was the lack of sufficient evidence to recommend the use of any oral medication or botulinum toxin treatment for reduction of dystonia.

Half a decade on from this publication, Fehlings et al. report upon the development and recommendations which have formed the second iteration of this pathway.^2^ For the development of this guideline, the authors have adopted the Grading of Recommendations, Assessment, Development and Evaluations (GRADE) approach,^3^ which has been applied to 10 specific ‘Population, Intervention, Comparator, Outcome’ (PICO) questions. Whilst the aims and intentions driving this endeavour remain laudable, and the methodology superior to the first iteration, the inescapable limitation remains the absence of available evidence to answer these questions.

Of the 10 questions asked, the strength of available evidence allowed only suggestions and not recommendations to be made. Consequently, the authors state this guideline is intended as a starting point for shared decision‐making, rather than a standard of care and as such it is to be entirely welcomed. The accompanying supplemental appendices represent a veritable treasure trove for those of us interested in tone management, outlining the data driving the decision‐making processes for these suggestions.

Compared to the 2018 report, the move to the GRADE approach has enabled the authors to arrive at suggestions for the use of oral medications. These suggestions will hopefully trigger some welcome debate amongst clinicians involved in the care of individuals with CP. Of the seven oral/enteral medications included, the use of baclofen, clonidine, and gabapentin are suggested in individuals with CP and generalized dystonia. In contrast, the routine use of trihexphenydil, benzodiazepines, levodopa, and medical cannabis are suggested against. All seven suggestions were based on very low certainty evidence.

Data are limited on the use of medications in the clinic to reduce dystonia in individuals with CP. Taking the available data from the UK, Australia, and North America collectively, the recommendations of Fehlings et al. are not entirely concordant with current clinical practice.^4^, ^5^ Use of trihexyphenidyl (and to a lesser extent, diazepam and levodopa) is common, whilst clonidine use is less reported.

One of the top 10 research priorities for interventions in childhood neurological conditions as established by the recent British Paediatric Neurology Association Priority Setting Project was: ‘Which medications should be used, and in what sequence, in the management of muscle stiffness (hypertonia) in children and young people?’ The low level of evidence informing the recommendations of Fehlings et al. illustrate the steepness of the hill we are to collectively climb in answering this question. Key to this will be to recognizing that different medications are likely to be preferable in different scenarios and contexts (e.g. the ambulant child with predominant lower limb spasticity who hopes to improve independence compared to the child with severe bilateral dystonia for whom the main priorities are around reductions of daily pain), necessitating different outcome measures and adjunct therapeutic support. A concerted effort will be required across the clinical and research community if we are to bridge the gap from suggestions to recommendations in future guidelines.

## Data Availability

Not required.
